# Comparative structure analyses of cystine knot-containing molecules with eight aminoacyl ring including glycoprotein hormones (GPH) alpha and beta subunits and GPH-related A2 (GPA2) and B5 (GPB5) molecules

**DOI:** 10.1186/1477-7827-7-90

**Published:** 2009-08-31

**Authors:** Eva Alvarez, Claire Cahoreau, Yves Combarnous

**Affiliations:** 1 Institut National de la Recherche Agronomique (INRA), Centre National de la Recherche Scientifique (CNRS), Unit « Physiologie de la Reproduction et des Comportements », 37380 Nouzilly, France

## Abstract

**Background:**

Cystine-knot (cys-knot) structure is found in a rather large number of secreted proteins and glycoproteins belonging to the TGFbeta and glycoprotein hormone (GPH) superfamilies, many of which are involved in endocrine control of reproduction. In these molecules, the cys-knot is formed by a disulfide (SS) bridge penetrating a ring formed by 8, 9 or 10 amino-acid residues among which four are cysteine residues forming two SS bridges. The glycoprotein hormones Follicle-Stimulating Hormone (FSH), Luteinizing Hormone (LH), Thyroid-Stimulating Hormone (TSH) and Chorionic Gonadotropin (CG) are heterodimers consisting of non-covalently associated alpha and beta subunits that possess cys-knots with 8-amino-acyl (8aa) rings. In order to get better insight in the structural evolution of glycoprotein hormones, we examined the number and organization of SS bridges in the sequences of human 8-aa-ring cys-knot proteins having 7 (gremlins), 9 (cerberus, DAN), 10 (GPA2, GPB5, GPHα) and 12 (GPHβ) cysteine residues in their sequence.

**Discussion:**

The comparison indicated that the common GPH-alpha subunit exhibits a SS bridge organization ressembling that of DAN and GPA2 but possesses a unique bridge linking an additional cysteine inside the ring to the most N-terminal cysteine residue. The specific GPHbeta subunits also exhibit a SS bridge organization close to that of DAN but it has two additional C-terminal cysteine residues which are involved in the formation of the "seat belt" fastened by a SS "buckle" that ensures the stability of the heterodimeric structure of GPHs. GPA2 and GPB5 exhibit no cys residue potentially involved in interchain SS bridge and GPB5 does not possess a sequence homologous to that of the seatbelt in GPH β-subunits. GPA2 and GPB5 are thus not expected to form a stable heterodimer at low concentration in circulation.

**Summary:**

The 8-aa cys-knot proteins GPA2 and GPB5 are expected to form a heterodimer only at concentrations above 0.1 microM: this would be consistent with a short-term paracrine role but not with an endocrine role after dilution in circulation. Consequently, GPA2 and GPB5 could exert separate endocrine roles either during development and/or during adult life of both vertebrates and invertebrates.

## Background

**Figure 1:**
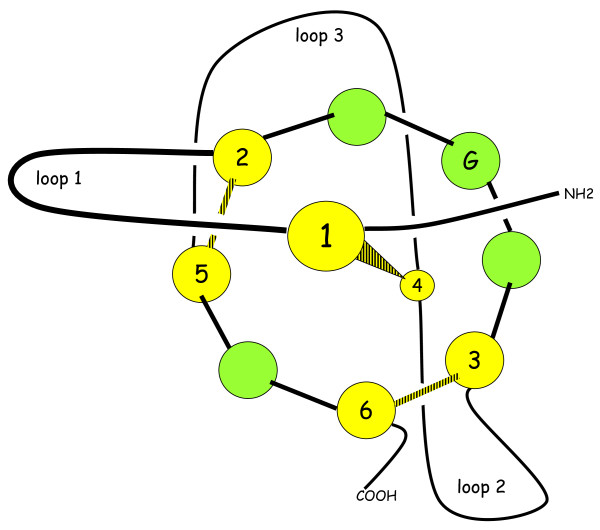
**Schematic view of the structure of 8-aa-ring cystine-knot proteins**. The six cysteines belonging to the cystine-knot are shown in yellow in this scheme and in the tables in additional files 1, 2 and 3. The remaining 4 amino-acids belonging to the 8-aa-ring are shown in green here as well as in the tables. G stands for the glycine residue that is conserved in all 8-aa-rings of cys-knot proteins. The black and yellow segments represent the three disulfide bridges forming the cystine-knot. The size of the ring is exagerated compared to that of the loops for the sake of clarity.

In cystine-knot (cys-knot) proteins, three disulfide (SS) bonds are arranged in such a way that one SS bond passes through the ring formed by two other SS bridges and the interconnecting peptide backbone [1,2]. The six cysteine residues forming three knotted SS bonds are numbered 1 to 6 from the N-terminus along the polypeptide sequence of these proteins and the SS bridge arrangements are always beween cys residues 1-4, 2-5 and 3-6 (figure 1). Nevertheless, cystine knot proteins belong to two large superfamilies named knottins [3,4] and cystine-knot Growth Factor related proteins [5]. In knottins it is the 3-6 bridge that passes through the frame formed by the two other SS bridges (1-4 and 2-5) whereas in cys-knot Growth Factor related proteins, it is the 1-4 bridge that passes through the frame formed by SS bridges (2-5 and 3-6) [3].

Cys-knot Growth Factor related proteins are secreted and act as growth factors (TGFβ, PDGF), as morphogens (BMPs, gremlins, DANs, Cerberus) and/or as hormones (glycoprotein hormones: LH, FSH, CG, TSH). Structurally, cys-knot Growth Factor related proteins have been classified, on the basis of phylogenetic analysis, as a function of the number of amino acid residues forming the ring i.e. 8-aa-, 9-aa- or 10-aa-ring [6]. Because of our interest in the structure-function relationships of glycoprotein hormones that are cys-knot proteins with 8-aa-ring, we more closely examined and compared the distribution of cysteine residues along the amino acid sequences of all 8-aa-ring cys-knot proteins. This comparison of the arrangements of cysteine residues and SS bridges in the amino acid sequences of 8-aa-ring cys-knot proteins was initiated in order to distinguish the specific structural features of the GPA2 and GPB5 molecules compared to glycoprotein hormones (GPH) subunits and to other 8aa-ring cys-knot proteins. Indeed GPA2 and GPB5 genes have been identified by genome sequence analysis in human [7] and other species [8] on the basis of their homology with GPH α and β subunits respectively. Because of this it was assumed that like GPH α and β subunits, GPA2 and GPB5 formed a heterodimer but this heterodimer was evidenced by SDS-PAGE only after chemical crosslinking of co-expressed recombinant GPA2 and GPB5 with disuccinimidyl suberate [7]. In the present study, we compare the arrangements of cysteine residues and SS bridges in GPA2, GPB5, GPH α- and β-subunits as well as other 8-aa-ring cys-knot proteins in order to support or to challenge the hypothesis that GPA2 and GPB5 can form a heterodimer in physiological situation.

## Discussion

Phylogenetic analyses of cys-knot proteins show that the 8-aa-ring proteins are more closely related to each other than to the 9-aa- and 10-aa ring subfamilies [6]. This gives support to the division of cys-knot Growth Factor related proteins into three subfamilies based on ring size and justifies the present analysis taking into account only 8-aa ring cys-knot proteins. The comparison of the arrangements of cysteine residues and SS bridges in the amino acid sequences of cys-knot proteins with 8-aa rings was initiated in order to distinguish the specific structural features of the GPA2 and GPB5 molecules compared to glycoprotein hormones (GPH) and to other 8-aa-ring cys-knot proteins.

### Cys distribution in 8- aa-ring cys-knot protein sequences

The sequences of the 8-aa-ring cys-knot proteins were retrieved from NCBI as indicated by Avsian-Kretchmer and Hsueh [6] plus those for GPA2 and GPB5 that were not considered in this previous study (hGPA2: NP 570125, hGPB5: NP 660154). The sequences of 8-aa-ring cys-knot proteins were aligned and their cysteine arrangements were compared as previously [6] and they are shown in the tables in additional files 1, 2 and 3.

The table in additional file 1 shows the comparison of cysteine arrangements in human 8-aa-ring cystine-knot proteins (see Additional file 1). The cysteine residues that form the cystine knot are numbered 1-6. Cysteine residues not belonging to the knot structure are found in loops 1, 2 and 3 of most cys-knot proteins and are noted I, II and III.

Cysteine residues 2, 3, 5, and 6 form the ring and are highlighted in pale yellow in additional file 1 and cysteine residues 1 and 4 form the knot (highlighted in darker yellow). The cystine ring is formed by the sequences from cys-2 to cys-3 and from cys-5 to cys-6 (non-cys residues highlighted in green in additional file 1).

The 8-aa cystine-knot proteins are presented in see additional file 1 according to their respective total numbers of cysteine residues. The hBMP-7 that like other BMPs possesses 7 cys residues, serves as the reference molecule since its crystalographic 3D-structure has been determined [9]. BMP-7 has only one cysteine residues in addition to the sixth residues forming the cystine-knot structure. This unpaired cysteine residue, shown with grey background in additional file 1, is believed to be involved in a symetrical SS bridge in homodimers of BMPs.

Gremlins and cerberus molecules both have 9 cysteine residues in their sequences. Therefore, they also have an odd number of cysteine residues meaning that they also have an unpaired cysteine inside their amino acid sequence. Compared to the BMP sequence, the additionnal SS bond links cysteine residues noted I and III in additional file 1 and belonging to loops 1 and 3 respectively.

The same SS bridge I-III is conserved in DAN, GPA2 and GPB5 which all have 10 cysteine residues in their sequences. The previously unpaired residue cys II in loop 2 of BMP, Grem and Cer is found in DAN and GPA2 to be linked to cys IV downstream of the ring in their C-terminal sequence. Consequently, in addition to the cystine knot, there are two additional SS bridges, one linking loops 1 and 3 and the other one linking loop 2 and C-terminus of these molecules with 10 cysteines. In GPB5, the previously unpaired residue cys II in loop 2 is absent and cys IV is not free but is bridged with an additional cys residue (cys V) only 7 aa downstream of it thus forming a very short loop.

GPHα also has 10 cysteine residues but it does not possess the SS bridge between cys I and cys III but instead exhibits an original bridge between an additional cysteine residue inside the 8-aa-ring that is linked to another additional cysteine at the N-terminus of the molecule. This feature in GPHα is unique among all 8-aa-ring cys-knot proteins. It is also noteworthy that the disappearance of the bridge between loops 1 and 3 in the GPHα subunit is paralleled by a shortening of these two loops compared to those in DAN, GPA2 and GPB5.

Human GPHβ chains possess 12 cysteine residues. Compared to GPB5, the two additional cys residues are located in loop 1 and at the terminus end respectively. These two cys residues have been shown to establish a bridge in hCG [1,2] as well as in FSH [10] and most probably in all GPHs. This bridge is known to be the "buckle" that fasten the "seat belt" formed by the C-terminal end of GPHβ subunits around their α-partners [11,12]. The cys IV-cys V bridge found in GPB5 is still present and has been described as a "tensioner" of the "seat belt" in GPHβ subunits [13,14].

Additional file 2 shows the arrangement of cysteines in GPB5 sequences from various vertebrate and invertebrate species compared to the human GPB5 that had been previously compared to the other 8aa-ring cys-knot proteins in additional file 1. It shows a great conservation of the general structure of these molecules throughout very distant species. Indeed, the distinctive features of hGPB5 compared to the other human 8-aa-ring knot-proteins (No cys II, presence of cys IV and V, no cys S) are strictly conserved in all vertebrate, ecdysozoan and lophotrochozoan species.

Additional file 3 shows the arrangement of cysteine residues in GPA2 sequences from various vertebrate and invertebrate species compared to the human GPA2 that had been previously compared to the other 8-aa-ring cys-knot proteins in additional file 1. The specific arrangement found in hGPA2 permits to differentiate putative GPA2 sequences in other species from other cys-knot proteins.

### Dimerization of 8-aa-ring cys-knot proteins

The 8-aa-ring cys-knot proteins exhibit 7, 9, 10 or 12 cysteine residues in their sequences. The proteins with odd numbers of cys residues (7 in BMP, 9 in Grem and Cer) exhibit one residue that is not involved in an intrachain SS bridge (shaded in grey in additional file 1) and which is suspected to establish an inter-chain SS bridge in homodimers.

In contrast, all 8-aa-ring cys-knot proteins with even numbers of cysteines (10 in DAN, GPA2, GPB5 and GPHα 12 in LHβ, FSHβ, TSHβ and CGβ) exhibit no unpaired cys residues and all of them are engaged in intra-chain SS bridges. Therefore, these molecules with even numbers of cysteines are not expected to form inter-chain SS bridges. Indeed this has been clearly shown for the heterodimeric glycoprotein hormones in which GPHα is non-covalently combined with either LHβ, FSHβ, TSHβ or CGβ. The heterodimeric structure of GPHs has been shown to be stabilized by a "seatbelt" sequence. The "seatbelt" in GPHβ subunits is "buckled" by a SS bridge between cysteine residues in the C-terminal region (cys110 in hCGβ) and one in loop 1 (cys26 in hCGβ), both marked SB (seatbelt buckle) in additional file 1.

These two cys residues are absent in the human GPB5 sequence (additional file 1) as well as in the GPB5 sequences of all other species (additional file 2). Therefore, no "seat belt" is expected in the GPB5 structure that could stabilize its association with GPA2. Numerous reports have stressed the importance of the seatbelt for GPH subunits heterodimerization. Nevertheless, a paper [15] published before the seatbelt structure was known [1,2] reported that *des *(101-145)hCGβ subunit cotransfected with bovine α subunit formed a biologically active heterodimer albeit at a lesser extent than the full-length β-subunit. The mutated *des *(101-145)hCGβ subunit lacks all C-terminal amino-acids beyond cys100 forming the seatbelt and has the same length as GPB5 (see. According to this report [15] the seatbelt might not be indispensable for GPH subunits heterodimerization and therefore GPA2 and GPB5 might combine in a heterodimer in spite of the absence of a potential seatbelt sequence in GPB5. Nevertheless, numerous papers in the literature indicate that the seatbelt and its fastening by a SS bridge are important for the stability of GPH heterodimers when secreted and diluted at very low concentrations. Indeed, when the cys residues in the "seat belt" are mutated [16] or reduced [17], a dramatic drop in heterodimer stability is observed. Indeed, the Kd of heterodimerization of GPH α and β subunits is approximately 10^-7^-10^-6 ^M [18,19] indicating that the presence of αβ heterodimer in the circulation at concentrations as low as 10^-11^-10^-9 ^M is possible only because of the slow rate of GPH dissociation [17] compared to their high rate of elimination [20]. Gonadotropin integrity *in vivo *is a kinetically regulated process [18] and the slow rate of GPH dissociation is due to the seatbelt. Indeed full dissociation is observed in less than five minute instead of 24 h when the disulfide bridge fastening the seatbelt is open in reducing condition (GSH/GSSG = 3) corresponding to that in endoplasmic reticulum [14,17].

GPB5 molecules do not possess the polypeptide regions corresponding to the seatbelt in glycoprotein hormones β-subunits (additional file 1). It is thus expected that they can indeed form a heterodimer with GPA2 with a Kd of approximately 10^-7^-10^-6 ^M like GPH subunits. Such a heterodimer would therefore exist only at concentrations above 3-30 μg/ml. In keeping with this observation, the recombinant heterodimer could be evidenced and purified when expressed at a very high concentration of 20 mg/l [21]. However, if the GPA2/GPB5 heterodimer is secreted in the circulation to exert an endocrine role, it should be present at micromolar concentration to retain its quaternary structure. It would probably have already been detected if present at such a high concentration.

In agreement with the absence of seatbelt in GPB5, the GPA2/GPB5 heterodimer was not evidenced by SDS-PAGE in contrast to GPH heterodimers [7]. Moreover, even after chemical cross-linking, only a faint spot was observed at the level expected for a dimer in SDS-PAGE whereas the majority of material was found at the monomer level [7].

### Expression patterns

It has been known for a long time that GPH α and β subunits readily form a heterodimer only when they are expressed in the same cell and associate at high concentration and under moderate reducing redox potential in endoplasmic reticulum to permit opening of the seatbelt SS buckle [12,14,16,17]. Because of the absence of seatbelt, heterodimerization of GPA2 and GPB5 should not require reducing redox potential but necessitates concentrations compatible with a Kd of more than 10^-7^M. It is thus expected that the two molecules are co-expressed in the same cell to allow dimerization. In fact, several papers report that GPA2 and GPB5 mRNAs exhibit different tissue expression patterns in various species [22]. In contrast, other papers claim that the two molecules are expressed together in pituitary corticotrophs [21] or in the eye and testis but not in the pituitary [23]. Another interesting observation is that GPA2 is expressed at much higher level and with higher tissue specificity than GPB5 that was found to be ubiquitarily expressed at low level both in amphioxus [22] and in rat and bovine (Haj Hassan M. *et al*., in preparation).

### Bioactivities of GPA2/GPB5 heterodimers

It is indeed important to ascertain that these molecules are not mere variants of other molecules before looking for their proper specific biological functions. Because of their homology with GPHs α and β-subunits respectively, GPA2 and GPB5 have been considered from the beginning to form a heterodimeric structure. In the princeps paper, heterodimerization was only observed after treatment with bifunctional reagent and was very partial even in these conditions (fig three in [7]). As this chemically cross-linked heterodimer was found to exhibit thyrotropic activity, it was named "thyreostimulin" [7].

A strong point in the *princeps *paper concerning GPA2 and GPB5 [7] is that the authors observed thyroid-stimulating activity for the cross-linked GPA2-GPB5 heterodimer. Like LH and FSH receptors, TSH receptor is a seven-transmembrane span receptor with a very large extracellular domain (ECD) that is responsible for high-affinity specific hormone binding. The three receptors exhibit strong homologies and constitute a well-defined superfamily. [24]. Nevertheless, in contrast to LH and FSH receptors, the TSH receptor undergoes proteolytic maturation at the hinge between its transmembrane domain and ECD and the two domains remain associated thanks to a SS bridge [25,26]. Also, in contrast to LH and FSH receptors, TSH receptor has been shown to be activable not only by its cognate hormone TSH but also by desialylated hCG [27,28] as well as by anti-TSH receptor antibodies [29] as first observed in hyperthyroidic diseases. It thus appears that the TSH receptor can be more readily stimulated by ligands other than its cognate hormone than LH and FSH receptors. It would thus be crucial that the GPA2/GPB5 heterodimer can be biochemically characterized to substantiate the functional properties that have been attributed to it [7,21,23,30-32].

The GPA2/GPB5 heterodimer under the name of Corticotroph-derived Glycoprotein Hormone (CGH) has also been described to possess anti-inflammation activity and to potentiate glucocorticoid action [33]. More recently, expression patterns of GPA2 and GPB5 were described during development [22] or in the adult [34,35] in amphioxus and Dos Santos *et al *[22] evidenced distinct expression patterns for the two molecules.

Taking all these information into account, it is reasonable to hypothesize that GPA2 and GPB5 might be either individually secreted or not remain as a heterodimer when secreted from the same cells when diluted in the bloodstream. GPA2 and GPB5 might thus play individual roles either in the adult or during development like other 8-aa-ring cys-knot proteins such as DAN, Cer or Grem [36]. In this respect, it is interesting to stress that free GPH-α subunit has been shown to exert a stimulatory role on lactogenic cells differenciation during pituitary development [37-40]. Potential roles for GPA2 and GPB5 monomers should also be searched [36].

## Conclusion

The present comparative analysis of 8-aa-ring cys-knot proteins raises arguments either supporting or challenging the view that the glycoprotein hormone subunits related molecules GPA2 and GPB5 really form a functional heterodimer *in vivo*. Further studies, in particular biochemical identification of the dimer and isolation of natural GPA2, GPB5 molecules and/or the GPA2/GPB5 dimer are indispensable to understand the biological role(s) of these molecules that are conserved in both vertebrates and invertebrates.

## Abbreviations

BMP: Bone-Morphogenic Protein; Cer: Cerberus (antagonist of BMP and Nodal); CG: Chorionic Gonadotropin; CGH: Corticotroph-derived Glycoprotein Hormone (= Thyrostimulin); DAN: Differential screening-selected gene Aberrative in Neuroblastoma (antagonist of BMP-7); FSH: Follicle-Stimulating Hormone or Follitropin; GPH: Glycoprotein hormone; GPA2: GPHα-related molecule; GPB5: GPHβ-related molecule; Grem: Gremlin (antagonist of BMP); LH: Luteinizing Hormone or Lutropin; OGH: Orphan Glycoprotein Hormone (= GPB5); TSH: Thyroid-Stimulating Hormone or Thyrotropin.

## Competing interests

The authors declare that they have no competing interests.

## Authors' contributions

All three authors contributed equally to sequence analyses and discussion of the data and literature. YC wrote the manuscript that was discussed and amended with EA and CC. All authors read and approved the final manuscript.

## Supplementary Material

Additional file 1 **Alignments of cysteines and disulfide bridges in 8-aa-ring Growth Factor-related molecules**. Table showing the alignments of cysteines and disulfide bridges in 8-aa-ring Growth Factor-related molecules Click here for file

Additional file 2 **Alignments of cysteines and disulfide bridges in GPB5 from various species**. Table showing the alignments of cysteines and disulfide bridges in GPB5 from various species Click here for file

Additional file 3 **Alignments of cysteines and disulfide bridges in GPA2 from various species**. Table showing alignments of cysteines and disulfide bridges in GPA2 from various species Click here for file
