# Supplementary material for: Comparative structure analyses of cystine knot-containing molecules with eight aminoacyl ring including glycoprotein hormones (GPH) alpha and beta subunits and GPH-related A2 (GPA2) and B5 (GPB5) molecules
Source: Reprod Biol Endocrinol. 2009 Aug 31;7:90. doi: 10.1186/1477-7827-7-90 (PMC3224965; doi:10.1186/1477-7827-7-90)
Supplement: Additional file 1 — Alignments of cysteines and disulfide bridges in 8-aa-ring Growth Factor-related molecules. Table showing the alignments of cysteines and disulfide bridges in 8-aa-ring Growth Factor-related molecules [file 1477-7827-7-90-S1.pdf]

Table 1. : *Alignements of cysteines and disulfide bridges in 8-aa-ring Growth Factor-related molecules*

	N-ter		k	loop 1						ring					loop 2			k	loop 3			ring				C-ter				
			**1**			**I**		**SB**		**2**				**3**		**II**		**4**		**III**		**5**		**6**		**IV**		**V**		**SB**
hBMP $_7$			*C*				28			*C*	1	*G*	1	*C*	31	*C*		*C*		31		*C*	1	*C*						
TGFβ $_9$	*C*	7	*C*	*C*			27			*C*	1	*G*	1	*C*	28	*C*		*C*		30		*C*	1	*C*						
GDF8 $_9$	*C*	8	*C*	*C*			26			*C*	1	*G*	1	*C*	25	*C*		*C*		31		*C*	1	*C*						
hGrem $_9$			*C*	13		*C*		9		*C*	1	*G*	1	*C*	18	*C*	2	*C*	13	*C*	17	*C*	1	*C*						
hCer1 $_9$			*C*	13		*C*		9		*C*	1	*G*	1	*C*	15	*C*	2	*C*	13	*C*	15	*C*	1	*C*						
hDAND1 $_{10}$			*C*	13		*C*		9		*C*	1	*G*	1	*C*	18	*C*	2	*C*	13	*C*	18	*C*	1	*C*	2	*C*				
hGPA2 $_{10}$			*C*	16		*C*		8		*C*	1	*G*	1	*C*	26	*C*		*C*	13	*C*	15	*C*	1	*C*	2	*C*				
hGPB5 $_{10}$			*C*	13		*C*		9		*C*	1	*G*	1	*C*	19			*C*	14	*C*	15	*C*	1	*C*	2	*C*	6	*C*		
hGPHα $_{10}$	*C*	2	*C*				17			*C*	1	*G*	*C*	*C*	26	*C*		*C*		22		*C*	1	*C*	2	*C*				
hGPHβ $_{12}$			*C*	13		*C*	2	*C*	7	*C*	1	*G*	1	*C*	18			*C*	14-16	*C*	15	*C*	1	*C*	2	*C*	6	*C*	9	*C*
hCGβ number.			*9*			*23*		*26*		*34*				*38*				*57*				*88*		*90*		*93*		*100*		*110*
																												seatbelt		

8-membered Ring : C2-1-G-1-C3-C6-1-C5-C2
perforing cystine : C1-C4
- red hyphens represent SS bridges
