# Supplementary material for: Comparative structure analyses of cystine knot-containing molecules with eight aminoacyl ring including glycoprotein hormones (GPH) alpha and beta subunits and GPH-related A2 (GPA2) and B5 (GPB5) molecules
Source: Reprod Biol Endocrinol. 2009 Aug 31;7:90. doi: 10.1186/1477-7827-7-90 (PMC3224965; doi:10.1186/1477-7827-7-90)
Supplement: Additional file 2 — Alignments of cysteines and disulfide bridges in GPB5 from various species. Table showing the alignments of cysteines and disulfide bridges in GPB5 from various species [file 1477-7827-7-90-S2.pdf]

Table 2. Alignements of cysteines and disulfide bridges in GPB5 from various species

			k	loop 1			ring					loop 2	k	loop 3			ring			C-term			
			1		I		2				3		4		III		5		6		IV		V
Chordate	*human*		C	13	C	9	C	1	G	1	C	19	C	14	C	15	C	1	C	2	C	6	C
	bos		C	13	C	9	C	1	G	1	C	19	C	14	C	15	C	1	C	2	C	6	C
	B florid		C	14	C	9	C	1	G	1	C	19	C	14	C	15	C	1	C	2	C	6	C
Ecdysozoa	*culex*		C	18	C	8	C	1	G	1	C	19	C	14	C	12	C	1	C	2	C	6	C
	anophel		C	18	C	8	C	1	G	1	C	19	C	14	C	12	C	1	C	2	C	6	C
	aedes		C	18	C	8	C	1	G	1	C	19	C	14	C	12	C	1	C	2	C	6	C
	droso		C	18	C	8	C	1	G	1	C	19	C	14	C	12	C	1	C	2	C	6	C
	bombyx		C	18	C	8	C	1	G	1	C	19	C	14	C	12	C	1	C	2	C	6	C
Loph	*aplysia*		C	23	C	8	C	1	G	1	C	19	C	14	C	15	C	1	C	2	C	6	C
	capitela		C	22	C	8	C	1	G	1	C	19	C	14	C	12	C	1	C	2	C	6	C
