# Supplementary material for: Comparative structure analyses of cystine knot-containing molecules with eight aminoacyl ring including glycoprotein hormones (GPH) alpha and beta subunits and GPH-related A2 (GPA2) and B5 (GPB5) molecules
Source: Reprod Biol Endocrinol. 2009 Aug 31;7:90. doi: 10.1186/1477-7827-7-90 (PMC3224965; doi:10.1186/1477-7827-7-90)
Supplement: Additional file 3 — Alignments of cysteines and disulfide bridges in GPA2 from various species. Table showing alignments of cysteines and disulfide bridges in GPA2 from various species [file 1477-7827-7-90-S3.pdf]

Table 3. Alignements of cysteines and disulfide bridges in GPA2 from various species

		k	loop 1			ring					loop 2		k	loop 3			ring			C-term		
		**1**		**I**		**2**				**3**		**II**	**4**		**III**		**5**		**6**		**IV**	
hGPA2		*C*	16	*C*	8	*C*	1	*G*	1	*C*	26	*C*	*C*	13	*C*	15	*C*	1	*C*	2	*C*	
mamals		*C*	16	*C*	8	*C*	1	*G*	1	*C*	26	*C*	*C*	13	*C*	15	*C*	1	*C*	2	*C*	
branch.		*C*	13	*C*	9	*C*	1	*G*	1	*C*	26	*C*	*C*	13	*C*	14	*C*	1	*C*	2	*C*	
anophel.		*C*	13	*C*	9	*C*	1	*G*	1	*C*	26	*C*	*C*	13	*C*	14	*C*	1	*C*	2	*C*	
culqu		*C*	13	*C*	9	*C*	1	*G*	1	*C*	26	*C*	*C*	13	*C*	14	*C*	1	*C*	2	*C*	
drosoph.		*C*	13	*C*	9	*C*	1	*G*	1	*C*	30	*C*	*C*	13	*C*	14	*C*	1	*C*	2	*C*	
bombyx		*C*	13	*C*	9	*C*	1	*G*	1	*C*	23	*C*	*C*	13	*C*	14	*C*	1	*C*	2	*C*	
